# Commentary: Regression Models—Efforts Are Required to Improve Statistical Practice and Teaching

**DOI:** 10.1002/sim.10341

**Published:** 2025-06-24

**Authors:** Willi Sauerbrei, Federico Ambrogi, Riccardo de Bin, Anne‐Laure Boulesteix, Els Goetghebeur, Marianne Huebner

**Affiliations:** ^1^ Institute of Medical Biometry and Statistics, Faculty of Medicine and Medical Center University of Freiburg Freiburg Germany; ^2^ Department of Clinical Sciences and Community Health University of Milan Milan Italy; ^3^ Department of Mathematics University of Oslo Oslo Norway; ^4^ Institute for Medical Information Processing, Biometry and Epidemiology, Faculty of Medicine Ludwig Maximilian University of Munich Munich Germany; ^5^ Department of Applied Mathematics, Computer Science and Statistics Ghent University Ghent Belgium; ^6^ Department of Statistics and Probability Michigan State University East Lansing Michigan USA

In their paper “*On the Uses and Abuses of Regression Models: A Call for Reform of Statistical Practice and Teaching*” Carlin and Moreno‐Betancur [1] engagingly illustrate issues in the applications of regression models. They consider naïve adjustment of covariates or misinterpretation of regression coefficients, adding three well‐chosen examples and a brief review of observational studies published in mainstream clinical research journals. One correctly points out that a clear definition of the research objective is often lacking, and encourage researchers to distinguish a descriptive, predictive, or causal purpose. The authors argue that teaching and training in statistical practice needs to be reformed to better prepare for correct application, evaluation, and interpretation of regression models. This includes a structured approach to specify the research question in terms of estimands, consider sampling and measurement issues, and pre‐specify an analysis plan. Researchers may be tempted to analyze data without carefully thinking about one or all three of these steps.

We appreciate these messages by the authors. The title is very general while the paper concentrates mainly on the teaching of statisticians in epidemiological methodology and ignores that the use and importance of regression models is much broader. Weaknesses identified in the clinical studies of the review are not only the result of bad teaching of regression models but can also result from a combination of reasons. These include insufficient knowledge about advantages and disadvantages of competing approaches (guidance is missing). Statistical analysis plans and their registration are more an exception than a rule for observational studies, and inadequate adherence to reporting guidelines.

As members of the STRATOS initiative our efforts are to provide accessible and evidence‐based guidance for key topics in the design and analysis of observational studies (https://www.stratos‐initiative.org). Below, we discuss several points which we believe to be important complements in teaching and practice.

## 1 There Is a Gap Between Teaching Regression Models and Applying Them Appropriately in Practice

A distinction not fully addressed in the paper, is the one between (bio‐) statisticians and users of statistical methods. Regression models are not only taught in statistics programs but also in domain sciences. Sometimes the only exposure to regression models that students experience is in software courses or workshops. These may focus only on technical aspects without emphasis on formulating a well‐defined research question or a careful consideration of data properties. It is important that users understand the key messages from model results and are familiar with the basic concepts and relevant issues of regression models.

Core properties of the regression model and its performance under different sets of assumptions play their role in a variety of settings. It deserves a dedicated teaching module. Evaluating the regression performance or impact on results when assumptions are not satisfied is an important aspect. However, there is a gap between teaching these topics, mindful of the relevance of statistical analysis plans, practicing transparent and reproducible reporting and the actual practice by analysts to adhere to these recommendations. Learners may require different formats of engagement and delivery to truly absorb and apply the teachings. This needs future work.

The work by the STRATOS initiative (https://www.stratos‐initiative.org) aims to fill a void to provide ongoing support to researchers and analysts with explanations of best practices illustrated with practical applications and examples and stay up‐to‐date with recent advances in statistics methodology [2].

## 2 Initial Data Analysis Needs to Be Included in a Roadmap for Analyses

Even when the research objectives are clear, statistical analysis plans often assume that a “clean” dataset is available. The roadmap to analysis proposed by the authors does include a consideration of sampling and measurement issues, but this does not address the full extent of considering the relevance of the data collection for the research question, the “zeroth problem” [3]. Pre‐specifying the analysis plan for estimation of the target parameters may not anticipate that data does not conform to expectations [4]. Analysis plans should include a systematic process of initial data analysis (IDA) which is a crucial step in providing reliable information about data properties to answer the research question and correct interpretation of regression coefficients [5]. The importance of IDA and implications for teaching has long been acknowledged [6] but is still often ignored. IDA is a prerequisite to all types of analyses. IDA may confirm the appropriateness of the intended analysis or reveal that it is necessary to update the modeling strategy to be able to interpret the results correctly [7]. Examining the data while abstaining from evaluating associations of outcome and covariates is often necessary to make appropriate modeling choices while not giving way to the temptation of selective reporting/fishing for significance. A possible outcome may even be that IDA is sufficient without further modeling [6].

## 3 The Practice of Reporting Guidelines Should Be Included in Teaching

The authors of the paper correctly pointed out that reporting a table with the estimates of the regression coefficient parameters is not sufficient to interpret results. Presenting tables with putative risk factors represents only part of the information collected to support an (causal) interpretation, but it is typically needed. Similarly, a table showing estimated coefficients of a predictive model provides an explanation of the model's functioning and does not necessarily imply that the included covariates are the (only) useful ones. The entire modeling process needs to be presented for readers to evaluate results. We disagree with the authors' claim that the emphasis on “goodness of fit” and on methods for model checking are a “reinforcement of the true model myth.” We see them as necessary and important tools for checking model assumptions. It may avoid strong bias, give additional insight and/or direct the statisticians to alternative analysis approaches as needed.

Unfortunately, the importance of transparent reporting and the necessity to provide guidance about presenting regression models is hardly mentioned in the paper. At the end authors cite the reporting guidelines CONSORT, TRIPOD, and TARGET, but the importance of reporting guidelines is not stressed. In another context authors cite Lesko et al. [8], who propose a framework for descriptive epidemiology. Reports of such studies should include 19 Items. It is a step in the right direction but reporting of statistical methods (items 12 and 16) does not require reporting all analyses with every relevant detail. In the explanation and elaboration paper of the Reporting Recommendations for Tumor Marker Prognostic Studies (REMARK), Altman et al. [9] stressed the importance to report all analyses and proposed the two‐part REMARK profile which summarizes key aspects, especially the derivation of the sample and details of all analyses planned and performed. Creating such profiles for 15 prognostic factor studies, Sauerbrei et al. [10] illustrated that structured reporting helps to get an overview of all analyses conducted, including checking assumptions and sensitivity analyses, and to identify limitations of analyses and results. Integrating such reporting guidance in the teaching practice is needed for future authors to provide clear explanations of the modeling choices and establish trust in the results. This is also one of the main concerns about the use of AI in medicine, that motivates the tremendous effort in research to explain black‐box Machine Learning models [11].

## 4 The Purpose of Regression Modeling Tasks Can Be More Complex Than Three Types

The authors argue that the purpose of analysis tasks in epidemiology research falls into three distinct categories: descriptive, predictive, causal. This is a helpful concept to initiate discussions between statisticians and domain expert collaborators. Regression coefficients may only be relevant for some statistics tasks but not others.

However, the situation is more complex. Causal inference tasks use regression models for various purposes each with distinct requirements, results, or validation [12]. For example, adding baseline covariates to estimate the effect of a treatment variable in regression models may increase the precision of the effect estimate in randomized trials. Observational studies require covariate adjustments to address confounding or handle effect modification when estimating an average treatment effect. The expected outcomes can then be standardized over the covariate distribution of a target population of interest. In contrast, the propensity score, which predicts treatment from baseline confounders, may simply aim for covariate balance in the treated and untreated groups after inverse weighting. Checking that the balance was achieved will confirm model performance for this purpose.

An important distinction between an “area where little is known” and one “where much is known” is made by the authors at the beginning of the article but it was not further considered. This aspect is relevant in all areas where empirical data is collected to gain first insights in the relevance of various factors. For example, the concept needs to be broader for studies at earlier stages, a topic discussed in the context of prognostic factor studies [13] and further extended in the PROGRESS (Prognosis research strategy) framework [14].

Often, little is also known when planning an analysis of high‐dimensional data, making the arguments in the paper difficult to apply. Causal reasoning in high dimensional analyses primarily focuses on discovering possible directed acyclic graphs using the large amount of available information [15].

## 5 Evidence‐Based Guidance and Neutral Comparison Studies Are Needed

The authors propose general principles for a reform of statistical practice and teaching. Recommendations to handle missing data, measurement error, variable and function selection, or time‐varying covariates are not the task of this paper, but importance of evidence‐based guidance is crucial. There are numerous options and competing approaches for many relevant topics and no analyst can be fully aware of advantages or disadvantages. For many topics “guidance” is proposed in various papers, but it cannot be formulated based on the limited and often biased comparisons of methods that are usually presented as part of articles suggesting new methods. Deriving evidence‐based guidance requires a comprehensive evaluation of new methods and neutral comparison studies to other methods to understand applicability and limitations in different scenarios [16, 17].

Such efforts are crucial for the development of reliable guidance and to help analysts navigate the complexity of modeling choices for appropriately and accurately addressing the research questions.

It is known that many analyses are conducted by researchers with a relatively weak statistical background and limited experience in using complex statistical methodology and software [2]. Consequently, guidance needs to depend on the level of statistical knowledge. STRATOS distinguishes between three levels.

In summary, the authors propose simple approaches to teaching regression models in the context of applied research questions. We suggest that several topics need to be included in more depth, such as initial data analysis and reporting. We stress the importance of guidance for the design and analysis of observational studies and caution that the three purposes of analysis tasks are not sufficient in many scenarios and that the analysis tasks are more complex.

## Conflicts of Interest

The authors declare no conflicts of interest.

## Data Availability

Data sharing not applicable to this article as no datasets were generated or analysed during the current study.

## References

[1] J. B. Carlin and M. Moreno‐Betancur , “On the Uses and Abuses of Regression Models: A Call for Reform of Statistical Practice and Teaching,” Statistics in Medicine 44, no. 13–14 (2025): 1–16. 10.1002/sim.10244. PMC12186762 40553044

[2] W. Sauerbrei , M. Abrahamowicz , D. G. Altman , S. L. Cessie , and J. Carpenter , “STRengthening Analytical Thinking for Observational Studies: The STRATOS Initiative,” Statistics in Medicine 33, no. 30 (2014): 5413–5432, 10.1002/sim.6265. 25074480 PMC4320765

[3] C. Mallows , “The Zeroth Problem,” American Statistician 52, no. 1 (1998): 1–9.

[4] D. L. DeMets , T. D. Cook , and K. A. Buhr , “Guidelines for Statistical Analysis Plans,” Journal of the American Medical Association 318, no. 23 (2017): 2301–2303, 10.1001/jama.2017.18954. 29260205

[5] M. Baillie , S. L. Cessie , C. O. Schmidt , L. Lusa , and M. Huebner , “Ten Simple Rules for Initial Data Analysis,” PLoS Computational Biology 18, no. 2 (2022): e1009819, 10.1371/journal.pcbi.1009819. 35202399 PMC8870512

[6] C. Chatfield , “The Initial Examination of Data,” Journal of the Royal Statistical Society. Series A (General) 148, no. 3 (1985): 214–231.

[7] G. Heinze , M. Baillie , L. Lusa , et al., “TG2 and TG3 of the STRATOS Initiative. Regression Without Regrets–Initial Data Analysis Is a Prerequisite for Multivariable Regression,” BMC Medical Research Methodology 24, no. 1 (2024): 178. 39117997 10.1186/s12874-024-02294-3 PMC11308558

[8] C. R. Lesko , M. P. Fox , and J. K. Edwards , “A Framework for Descriptive Epidemiology,” American Journal of Epidemiology 191, no. 12 (2022): 2063–2070, 10.1093/aje/kwac115. 35774001 PMC10144679

[9] D. G. Altman , L. M. McShane , W. Sauerbrei , and S. E. Taube , “Reporting Recommendations for Tumor Marker Prognostic Studies (REMARK): Explanation and Elaboration,” BMC Medicine 10, no. 1 (2012): 1–39, 10.1186/1741-7015-10-51. 22642691 PMC3362748

[10] W. Sauerbrei , T. Haeussler , J. Balmford , and M. Huebner , “Structured Reporting to Improve Transparency of Analyses in Prognostic Marker Studies,” BMC Medicine 20, no. 1 (2022): 184, 10.1186/s12916-022-02304-5. 35546237 PMC9095054

[11] V. Hassija , V. Chamola , A. Mahapatra , et al., “Interpreting Black‐Box Models: A Review on Explainable Artificial Intelligence,” Cognitive Computation 16, no. 1 (2023): 45–74, 10.1007/s12559-023-10179-8.

[12] E. Goetghebeur , S. L. Cessie , B. De Stavola , E. E. Moodie , and I. Waernbaum , “Formulating Causal Questions and Principled Statistical Answers,” Statistics in Medicine 39, no. 30 (2020): 4922–4948, 10.1002/sim.8741. 32964526 PMC7756489

[13] D. G. Altman and G. H. Lyman , “Methodological Challenges in the Evaluation of Prognostic Factors in Breast Cancer,” Breast Cancer Research and Treatment 52, no. 1‐3 (1998): 289–303, 10.1023/a:1006193704132. 10066088

[14] H. Hemingway , P. Croft , P. Perel , et al., “Prognosis Research Strategy (PROGRESS) 1: A Framework for Researching Clinical Out‐Comes,” BMJ 346 (2013): e5595. 23386360 10.1136/bmj.e5595 PMC3565687

[15] P. Bühlmann , P. Drineas , M. Kane , and M. van der Laan , Handbook of Big Data (Boca Raton: Chapman & Hall/CRC, 2016).

[16] A. L. Boulesteix , H. Binder , M. Abrahamowicz , and Sauerbrei W for the Simulation Panel of the STRATOS Initiative , “On the Necessity and Design of Studies Comparing Statistical Methods,” Biometrical Journal 60, no. 1 (2018): 216–218. 29193206 10.1002/bimj.201700129

[17] G. Heinze , A. L. Boulesteix , M. Kammer , T. P. Morris , and White IR for the Simulation Panel of the STRATOS initiative , “Phases of Methodological Research in Biostatistics‐Building the Evidence Base for New Methods,” Biometrical Journal 66, no. 1 (2024): e2200222. 36737675 10.1002/bimj.202200222 PMC7615508

